# Beyond Water Oxidation: Hybrid, Molecular-Based Photoanodes for the Production of Value-Added Organics

**DOI:** 10.3389/fchem.2022.907510

**Published:** 2022-05-25

**Authors:** Mirco Natali, Andrea Sartorel, Albert Ruggi

**Affiliations:** ^1^ Department of Chemical Pharmaceutical and Agricultural Sciences (DOCPAS), University of Ferrara, Ferrara, Italy; ^2^ Dipartimento di Scienze Chimiche, Università di Padova, Padova, Italy; ^3^ Département de Chimie, Université de Fribourg, Fribourg, Switzerland

**Keywords:** artificial photosynthesis, molecular sensitizer, solar fuels, value-added organics, hybrid photoanode

## Abstract

The political and environmental problems related to the massive use of fossil fuels prompted researchers to develop alternative strategies to obtain green and renewable fuels such as hydrogen. The light-driven water splitting process (i.e., the photochemical decomposition of water into hydrogen and oxygen) is one of the most investigated strategies to achieve this goal. However, the water oxidation reaction still constitutes a formidable challenge because of its kinetic and thermodynamic requirements. Recent research efforts have been focused on the exploration of alternative and more favorable oxidation processes, such as the oxidation of organic substrates, to obtain value-added products in addition to solar fuels. In this mini-review, some of the most intriguing and recent results are presented. In particular, attention is directed on hybrid photoanodes comprising molecular light-absorbing moieties (sensitizers) and catalysts grafted onto either mesoporous semiconductors or conductors. Such systems have been exploited so far for the photoelectrochemical oxidation of alcohols to aldehydes in the presence of suitable co-catalysts. Challenges and future perspectives are also briefly discussed, with special focus on the application of such hybrid molecular-based systems to more challenging reactions, such as the activation of C–H bonds.

## Introduction

**FIGURE 1:**
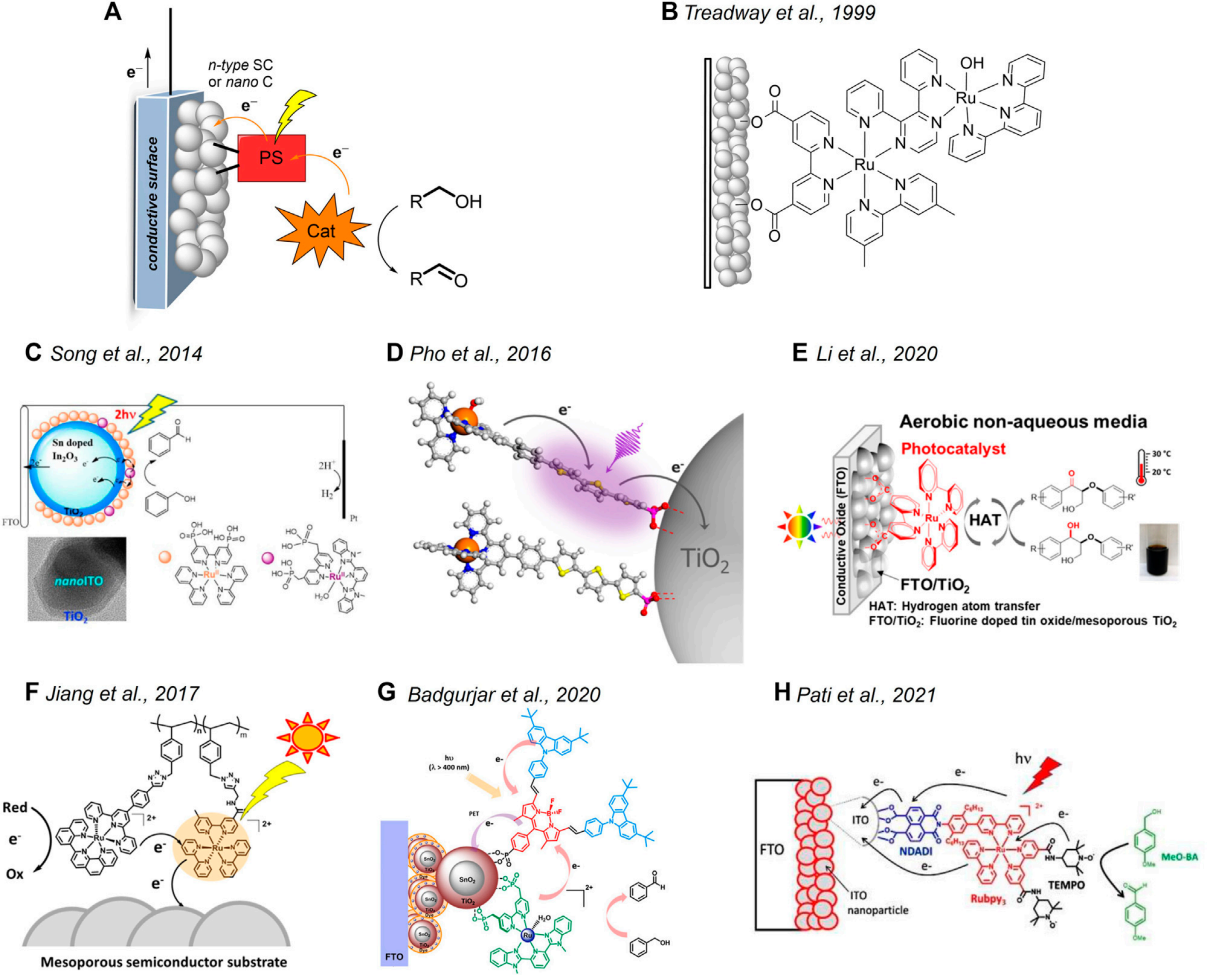
**(A)** Main components of a hybrid photoanode for alcohol oxidations and related light-driven processes (legend: SC = semiconductor, C = conductor, PS = photosensitizer, Cat = catalyst). **(B–H)** Hybrid systems based on Ru-derivatives discussed in *Systems Based on Ruthenium Complexes section*. **(C)** Reprinted with permission from Song et al. (2014). Copyright 2014 American Chemical Society. **(D)** Reprinted with permission from Pho et al. (2016). Copyright 2016 American Chemical Society. **(E)** Reprinted with permission from Li et al. (2020). Copyright 2020 American Chemical Society. **(F)** Reprinted with permission from Jiang et al. (2017). Copyright 2017 American Chemical Society. **(G)** Reprinted with permission from Badgurjar et al. (2020). Copyright 2020 American Chemical Society. **(H)** Reprinted with permission from Nikoloudakis et al. (2021). Copyright 2021 John Wiley and Sons.

Sunlight constitutes a virtually unlimited, widely available, and highly energetic resource. The utilization of solar energy to produce chemical fuels (e.g., hydrogen or CO_2_ reduction products) is undoubtedly one of the most promising strategies to circumvent the intrinsic limitations of sunlight (Field et al., 1998; Balzani et al., 2008; Schultz and Yoon, 2014; Lewis, 2016). Most of the methods developed so far have been focused on the light-driven water splitting process: the utilization of sunlight to break the molecule of water into hydrogen and oxygen. Generally speaking, this process requires three components: a molecule capable of harvesting sunlight (sensitizer) and two catalysts suitable for performing the oxidation of water to oxygen and the reduction of protons to hydrogen (Concepcion et al., 2012). Extensive investigations lead to the development of several efficient systems for light-triggered hydrogen production (Mazzeo et al., 2021), whereas water oxidation still constitutes a bottleneck issue. In fact, water oxidation usually requires strongly oxidizing conditions, which often results in the degradation and consequent inactivation of the catalysts (Hong et al., 2013; Macchioni, 2019). Moreover, the water oxidation process is a four-electron/four-proton process with the formation of a new O–O bond and is thus associated with low kinetics. Eventually, molecular oxygen does not constitute an interesting product from the application viewpoint since it is widely available in the air (Inoue et al., 2011). To circumvent these issues, researchers have recently started to investigate the possibility of coupling proton reduction with other chemical reactions. More in detail, two main processes have been performed to achieve this goal: waste photoreforming and photoredox catalysis on pure organic substrates to obtain value-added products (Reisner, 2019; Toe et al., 2021; Kim et al., 2022). In photoreforming, the proton reduction reaction is coupled with the oxidation of organic substrates such as waste, plastics, and pollutants, with the formation of either CO_2_ or value-added compounds (Cui et al., 2021; Luo et al., 2021). The main advantage of this strategy is the possibility of transforming environmentally harmful chemicals (which are costless and whose elimination is indeed a cost) into hydrogen and other fuels. However, the low solubility and/or the low concentration of the substrates pose a major kinetic challenge which constitutes the main drawback of this approach (Butburee et al., 2020; Nwosu et al., 2021; Toe et al., 2021; Uekert et al., 2021). Conversely, the coupling of proton reduction with photoredox catalysis aims at obtaining value-added organic products and fine chemicals starting from pure compounds such as alcohols, acids, aldehydes, and amines. Compared with photoreforming, photoredox catalysis requires pure starting materials, which might somehow reduce the cost-effectiveness of the process (Lhermitte and Sivula, 2019; Agosti et al., 2020; Tang and Sun, 2020; Qi et al., 2021). The vast majority of the systems reported so far both for photoreforming and photoredox catalysis are based on semiconductor nanoparticles, mostly used as suspensions. Several reviews have been recently published on this subject and will not be discussed here (Kampouri and Stylianou, 2019; Qi et al., 2021; Yuan et al., 2021; Casadevall, 2022). Material-based systems, either used as suspensions or electrodes, usually yield better performing and easy-to-handle devices, although at the expense of a more challenging mechanistic investigation and component tailoring, which are typical properties of systems based on organic or coordination compounds (Berardi et al., 2014; Zhou et al., 2015; Zhang and Sun, 2019; Qi et al., 2021). Systems including molecular species grafted on semiconductors and immobilized on photoelectrodes (hybrid systems) enable the merging of the best of both strategies. Such architectures could play an important role in photoredox catalysis, exploiting the tunability and the straightforward mechanistic investigation of the molecular systems coupled with the stability of semiconductors (Hennessey and Farràs, 2018; Wu et al., 2018). The related main components and the basic mechanistic steps are shown in Figure 1A. A sensitizer anchored onto the electrode is used as the light-harvesting unit to initiate the redox processes *via* electron injection. A catalyst, either attached to the electrode or in the solution, is then used to receive the oxidizing equivalents from the oxidized sensitizer in a stepwise manner and perform the transformation of the substrate. Injected electrons are collected in a dark cathode to accomplish the fuel-forming reaction (e.g., hydrogen generation). This field of research is still in its infancy: the very few reports which have appeared so far constitute the object of this article. In this mini-review, we discuss in detail some selected examples of hybrid systems used in oxidative photoredox catalysis with the aim of drawing conclusions which could orient the community toward the development of more efficient solutions. The oxidation of alcohols was chosen as the target reaction since it has been widely explored in recent literature in conjunction with fuel-forming reactions, for example, hydrogen evolution. The selected systems provide examples of different architectures, separately discussed on the basis of the active components. These include Ru-based systems (*Systems Based on Ruthenium Complexes section*) and noble-metal-free ones (*Systems Based on Noble-Metal-Free and Organic Sensitizers section*) grafted on electrodes based on either nanocrystalline n-type inorganic semiconductors (TiO_2_, SnO_2_, or related core-shell systems) or mesoporous conductors such as *nano-*ITO (Eftekhari et al., 2017). For the sake of comparison, the main photoelectrochemical results are summarized in Table 1.

**TABLE 1:** Relevant photoelectrochemical data of the systems discussed in *Systems Based on Ruthenium Complexes section* and *Systems Based on Noble-Metal-Free and Organic Sensitizers section* used in the oxidation of organic substrates.

Substrate/product (reference)	J^a^ (μA/cm^2^)	E (V)^b^	IPCE (%)	FE (%)	Stability^c^ (h)	Conditions
*Ru-based systems (* *Section 2* *)*						
Benzyl alcohol/benzaldehyde (Song et al., 2014)	∼200	0.2^d^	1 at 445 nm^e^	37	n.d.	*nano*ITO|TiO_2_, pH 4.5
Benzyl alcohol/n.d. (Jiang et al., 2017)	∼10	0.2	n.d.	n.d.	n.d.	TiO_2_, pH 4.6
Phenol/n.d. (Jiang et al., 2017)	∼10	0.2	n.d.	n.d.	n.d.	TiO_2_, pH 4.6
Benzyl alcohol/n.d. (Pho et al., 2016)	∼30	0.2	n.d.	n.d.	n.d.	TiO_2_, pH 4.35
Phenol/1,2-benzoquinone (Pho et al., 2016)	∼70	0.2	n.d.	n.d.	n.d.	TiO_2_, pH 4.35
2-phenoxy-1-phenylethanol/2-phenoxy-1-phenylethanone (Li et al., 2020)	∼90	0.8^f^	n.d.	91	80	TiO_2_, CH_3_CN, NHPI/lutidine
Benzyl alcohol/benzaldehyde (Badgurjar et al., 2020)	∼35	0.2	n.d.	n.d.	0.2	core-shell SnO_2_/TiO_2_, pH 4.65
4-methoxybenzyl alcohol/methoxybenzyl aldehyde (Nikoloudakis et al., 2021)	<180	0.45^f^	n.d.	80^g^	1	*Nano-*ITO, pH 10
*Organic and noble-metal-free based systems (* *Section 3* *)*						
4-methoxybenzyl alcohol/4-methoxybenzaldehyde (Nikoloudakis et al., 2021)	200	0.04^f^	2.6 at 430 nm	82^h^	1.5	TiO_2_, pH 8 or CH_3_CN/MeImd.
Benzyl alcohol/benzaldehyde (Bruggeman et al., 2021a)	400	0	n.d.	100	32	TiO_2_, CH_3_CN, TEMPO/LiTFSI
4-methylbenzyl alcohol/4-methylbenzaldehyde (Antón-García et al., 2022)	∼90	−0.27^i^	2 at 425 nm	87^j^	22	TiO_2_, pH 8
Benzyl alcohol/n.d. (Zhuang et al., 2020)	18	0.6	n.d.	n.d.	2	ITO, CH_3_CN, TEMPO.
Benzyl alcohol/n.d. (Volpato et al., 2021)	∼90	0.4	n.d.	n.d.	2	SnO_2_, CH_3_CN, NHS/pyridine

Abbreviations: NHPI = N-hydroxyphthalimide, MeImd: N-methylimidazole, LiTFSI = lithium bis(trifluoromethanesulfonyl)imide NHS = N-hydroxysuccinimide, n.d. = not determined.

**FIGURE 2:**
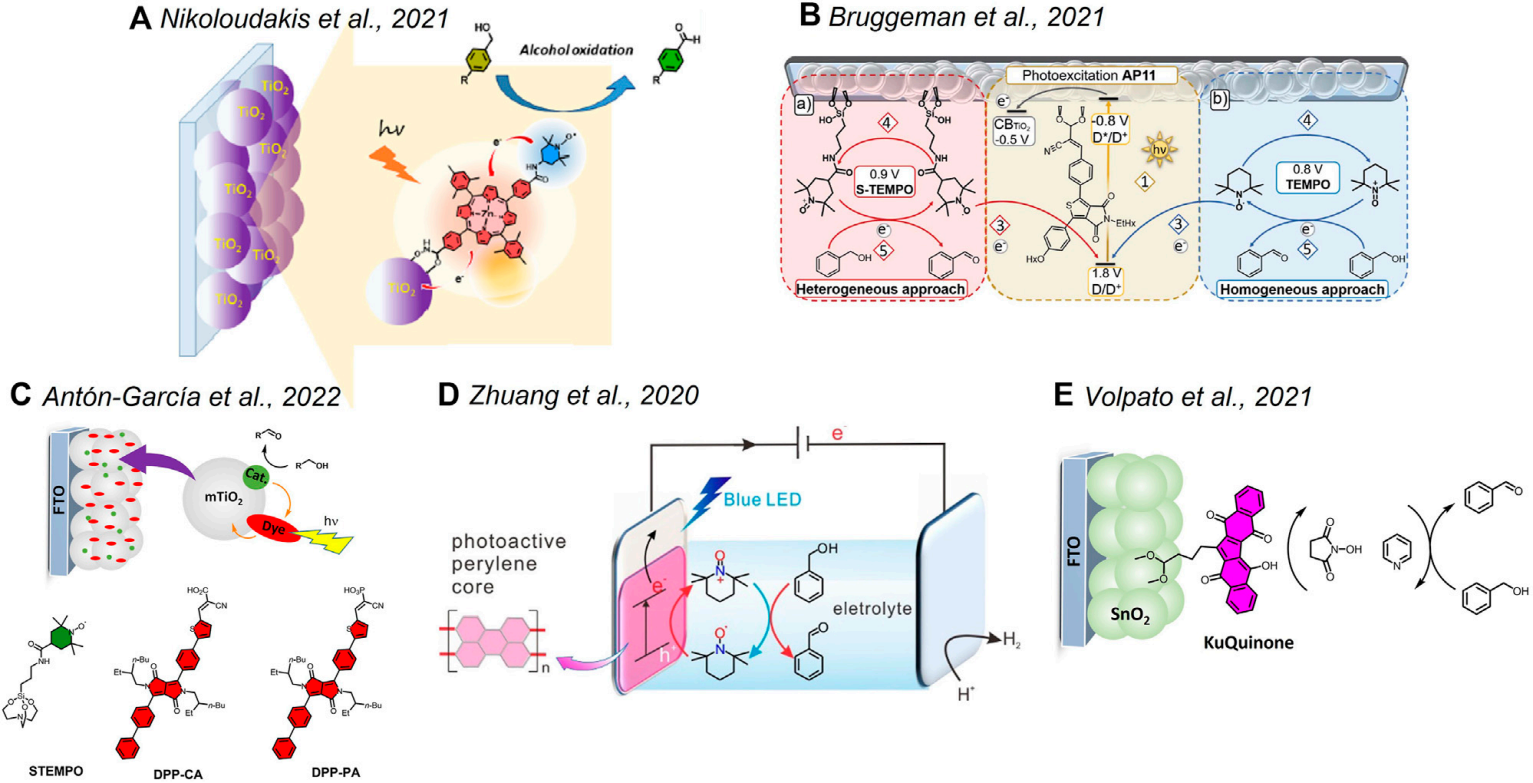
Hybrid systems discussed in Section 3 based on noble-metal–free and organic components. **(A)** Adapted with permission from Nikoloudakis et al. (2021). Copyright 2021 American Chemical Society. **(B)** Reprinted with permission from Bruggeman et al. (2021b). Creative Commons Attribution-Noncommercial 3.0 Unported License. **(D)** Adapted with permission from Zhuang et al. (2020). Copyright 2020 American Chemical Society.

a current density is expressed per geometric surface area;

b potential bias referenced *vs*. Ag/AgCl for a direct comparison of the systems;

c indicative time of activity as reported in the publication;

d originally reported *vs*. NHE and converted according to the formula E (V, *vs.* Ag/AgCl) = E (V, *vs*. NHE)—0.2;

e estimated using the known APCE, the dye loading, and the resulting LHE;

f originally reported *vs*. SCE and converted according to the formula E (V, *vs*. Ag/AgCl) = E (V, *vs*. SCE) + 0.04V;

g reported turnover number (TON) = 151;

h reported TON = 26;

i originally reported *vs*. RHE and converted according to the formula E (V, *vs*. Ag/AgCl) = E (V, *vs*. RHE)—0.2–0.0592 × pH;

j reported TON = 131 ± 22 (STEMPO) and TON = 853 ± 107 (DPP-CA, see Figure 2 for the structures).

## Systems Based on Ruthenium Complexes

The idea of using heterogenized ruthenium-based molecular species to accomplish light-driven oxidation reactions was first reported in 1999 by T. J. Meyer et al., who synthesized a dyad (Figure 1B) featuring a ruthenium polypyridine chromophore coupled with a ruthenium oxidation catalyst through a 2,3-bis(2-pyridyl)-pyrazine (dpp) bridge (Treadway et al., 1999). When attached to nanocrystalline TiO_2_, the dyad was capable of driving the conversion of 2-propanol to acetone on visible light irradiation with concomitant hydrogen evolution at the counter electrode. Although the performance of the system was not optimized (maximum current densities achieved were <1 μA/cm^2^), this work pioneered the use of heterogenized molecular components to perform oxidative organic transformation.

Following these premises, T. J. Meyer et al. reported the oxidation of benzyl alcohol using photoanodes based on either mesoporous TiO_2_ or core-shell *nano-*ITO|TiO_2_ nanoparticles functionalized with a chromophore and a catalyst based on ruthenium complexes (Figure 1C) (Song et al., 2014). Maximum photocurrent densities (J) of ∼60 (+0.2 V *vs.* NHE applied bias) and ∼200 μA/cm^2^ (+0.4 V *vs*. NHE applied bias) were measured in the presence of 0.1 M benzyl alcohol for the TiO_2_ and *nano-*ITO|TiO_2_ photoanodes, respectively. An absorbed photon-to-current conversion efficiency (APCE) of 0.36 and 3.7% was recorded for the two photoanodes. The improved activity of the core-shell system was attributed to the decreased charge recombination with respect to the sole TiO_2_. Photo electrolysis experiments showed the formation of benzaldehyde as the oxidation product with Faradaic yields of 26 and 37% for the TiO_2_ and *nano-*ITO|TiO_2_ photoanodes, respectively. For these systems, the photo-oxidation reaction is mediated by high-valent Ru^IV^=O species generated upon irradiation, after the electron injection of the chromophore unit into the oxide support and subsequent hole transfer to the catalyst. In a similar approach, Leem et al. prepared a polystyrene-based system comprising both ruthenium-based catalysts and sensitizers (Figure 1F) (Jiang et al., 2017). The deposition of such a polymeric assembly onto nanocrystalline TiO_2_ leads to active photoanodes for the light-driven oxidation of phenol and benzyl alcohol. Under 1 Sun irradiation and on the application of a +0.2 V versus Ag/AgCl potential bias, J in the order of ∼10 μA/cm^2^ were recorded in an acetate buffer solution at pH 4.6 in the presence of 12 mM phenol or 0.1 M benzyl alcohol.

In an attempt to replace the ruthenium-based chromophore with an organic molecular scaffold, Schanze et al. designed a molecular dyad (Figure 1D) featuring a terthiophene fragment as the sensitizing unit covalently linked to a ruthenium catalyst (Pho et al., 2016). When adsorbed onto mesoporous TiO_2_, the resulting photoanode was capable of promoting the visible light–driven oxidation of phenol mediated through a Ru^IV^=O intermediate. J in the order of ∼70 μA/cm^2^ was measured at 1 Sun illumination and +0.2 V versus SCE bias at pH 4.35 (acetate buffer) in the presence of 16 mM phenol. Long-term photoelectrolysis showed the formation of 1,2-benzoquinone as the dominant product. This photoanode was also active in the oxidation of benzyl alcohol, albeit with smaller J (∼30 μA/cm^2^ under identical conditions, 0.1 M substrate concentration) ascribed to the sluggish oxidation kinetics by the ruthenium catalyst. Similarly, T. J. Meyer et al. reported the use of core-shell SnO_2_/TiO_2_ photoanodes prepared by the co-sensitization of the semiconductor with phosphonate-derivatized BODIPY chromophores and a ruthenium catalyst (Figure 1G) (Badgurjar et al., 2020). On 1 Sun irradiation and +0.2 V *vs*. Ag/AgCl potential bias, J as large as 35 μA/cm^2^ was recorded in the acetate buffer solution at pH 4.65 in the presence of 0.1 M benzyl alcohol.

Parallel attempts at replacing the ruthenium catalytic unit with organic co-catalysts were also made. Leem et al. used a photoanode based on a classical ruthenium chromophore attached to mesoporous TiO_2_ (Figure 1E) for light-driven oxidation reactions in organic solvents mediated by a dissolved *N*-hydroxyphthalimide (NHPI) co-catalyst (Li et al., 2020). The almost quantitative (91%) photo-oxidation of a benzyl alcohol derivative to the corresponding ketone within ca. 20 h was accomplished under visible-light irradiation (2 suns) and +0.75 V vs. SCE-applied bias of an acetonitrile solution containing 5 mM NHPI, 5 mM 2,6-lutidine, and 2.5 mM substrate. The photoreaction involves the electron injection of the dye onto TiO_2_ upon photo-excitation followed by the proton-coupled oxidation of the NHPI in the solution to the *N*-oxyl PINO radical, which eventually promotes substrate conversion through a hydrogen atom transfer (HAT) mechanism. More interestingly, the hybrid system turns out to be effective in the photo-oxidation of natural lignin in the acetone solution, with measured J of ∼130 μA/cm^2^ under comparable experimental conditions. Odobel et al. reported the synthesis of a molecular triad based on a ruthenium tris (bipyridine) complex as the photosensitizer, connected on one side to the TEMPO alcohol oxidation catalyst and on the other side to a naphthalenedicarboxyanhydride dicarboximide (NDADI) electron acceptor (Figure 1H) (Nikoloudakis et al., 2021). When attached onto *nano-*ITO, the triad effectively mediates the oxidation of *para*-methoxybenzyl alcohol in a pH 10 carbonate buffer solution on visible light irradiation at a bias of +0.4 V versus SCE. A maximum Faradaic efficiency of ∼80% was recorded for the formation of *para*-methoxybenzaldehyde, suggesting the enhanced selectivity of the photocatalytic system. The system deactivates along 1 h photoelectrolysis due to partial leaching of the molecular triad from the ITO surface and the concomitant degradation of the ruthenium chromophore unit. Substrate activation occurs *via* electron transfer from the excited ruthenium dye to *nano-*ITO, likely mediated by the NDADI acceptor, followed by hole transfer to the TEMPO unit which promotes alcohol oxidation through a HAT mechanism, similar to the NHPI system previously discussed (Li et al., 2020).

## Systems Based on Noble-Metal–Free and Organic Sensitizers

Inspired by the natural photosystem II, porphyrinoid derivatives were considered viable alternatives to ruthenium polypyridine dyes in light-induced water oxidation cycles (Orbelli Biroli et al., 2019) and exploited in the design of photoanodes in combination with suitable catalysts (Moore et al., 2011; Poddutoori et al., 2015; Yamamoto et al., 2016).

The lower oxidation power required for the transformation of organics can open new opportunities for a plethora of porphyrin dyes which usually require ring functionalization to achieve potentials suitable for water oxidation. Within this framework, one elegant system was recently proposed by Odobel et al., combining a zinc porphyrin and a TEMPO catalyst onto a TiO_2_ semiconductor (Figure 2A) (Nikoloudakis et al., 2021). The system was successfully applied in the photoelectrochemical oxidation of benzyl alcohol derivatives in the presence of methyl imidazole as the base, with better performances observed in aqueous borate buffer with respect to the acetonitrile solution. J ∼ 200 μA/cm^2^ was observed at pH 8 at 0 V versus SCE, associated with a Faradaic yield of ∼82% for benzyl alcohol oxidation along a 2-h photo electrolysis. The incident photon-to-current conversion efficiency (IPCE) reached 2.6% at 430 nm, in correspondence with the Soret absorption. Transient absorption studies confirmed an ultrafast electron injection of the excited Zn porphyrin into the TiO_2_ conduction band with the formation of the porphyrin radical cation. A slow TEMPO→ZnPor^+^ electron transfer then occurs in the hybrid material, only partially competing with recombination, consistent with the low driving force for the process (0.1 eV) and the poor electronic coupling between the units. Interestingly, similar photoelectrochemical performances were also obtained by adding the TEMPO catalyst in a homogenous solution, avoiding the synthetic effort to bind it to the porphyrin chromophore. The aspect of heterogenizing the TEMPO catalyst for alcohol oxidation was also discussed by Reek et al. (Bruggeman et al., 2021a; Bruggeman et al., 2021b), in the development of photoanodes sensitized with thienopyrroledione-based organic dye (AP11, Figure 2B). The AP11 dye can be efficiently chemisorbed onto TiO_2,_ and electron injection from the excited state is energetically favorable by 0.3 eV. The AP11^+^/AP11 couple is characterized by a high oxidizing power (E = +1.80 V *vs.* NHE) and can promote the oxidation of TEMPO. Under optimized conditions, the photoelectrochemical systems are active toward the oxidation of benzyl alcohol to benzaldehyde with an almost quantitative Faradaic efficiency, characterized by J up to 400 μA/cm^2^ at 0 V versus Ag/AgCl, stable for up to 32 h at 50 mW/cm^2^ irradiation, although high concentrations of TEMPO and lithium bis(trifluoromethanesulfonyl)imide as a base were required (1.0 and 1.2 M, respectively, in acetonitrile) (Bruggeman et al., 2021a). To overcome this drawback, the authors proposed to anchor the TEMPO catalyst onto the TiO_2_ layer by exploiting a silatrane linker (Bruggeman et al., 2021b). However, the system performed worse (i.e., an order of magnitude lower J and a decreased stability) with respect to the one keeping TEMPO in the solution. The authors attributed the poor performance of the integrated dye/catalyst photoanodes to inefficient hole migration from the oxidized form of the dye to the TEMPO catalyst and to recombination events involving back-electron transfer from TiO_2_ to oxidized TEMPO^+^ species at the surface. A similar “fully heterogenous” approach was recently developed by Warnan and Reisner by chemisorbing diketopyrrolopyrrole dyes (through carboxylic or phosphonic acid anchoring groups) and a TEMPO catalyst onto TiO_2_ (Figure 2C) (Antón-García et al., 2022). Importantly, the dyes were designed for localizing the LUMO in the proximity of the anchoring group, thus favoring electron injection into the semiconductor. The photoanodes reached J up to ∼90 μA/cm^2^ for oxidation of 4-methyl-benzyl alcohol in borate buffer (pH 8) when applying +0.4 V versus RHE, with Faradaic yields in the range 80–100% and 60–90% when using the carboxylic and phosphonic anchoring groups, respectively. Under these conditions, the IPCE reached 2% at 425 nm and the electrodes showed good stability, retaining 50% of the activity after 12 h. Interestingly, a similar efficiency was also observed for the oxidation of 5-hydroxymethylfurfural to 2,5-diformylfuran (J up to ∼70 μA/cm^2^, FY up to 90%). The photoanodes were ultimately coupled with a formate dehydrogenase (FDH) enzyme–based cathode. Under bias-free conditions, the assembled two-compartment photoelectrochemical cell provides a stable J ∼ 30 μA/cm^2^ over the course of 6 h of irradiation, with a quantitative Faradaic yield for oxidation of 4-methyl-benzyl alcohol to aldehyde and a ∼74% Faradaic yield for CO_2_ reduction to formate.

Other organic dyes that were considered in the photoanodes for alcohol oxidations are perylene (onto ITO) (Zhuang et al., 2020) and polyquinoid Ku-quinone (onto mesoporous SnO_2_) (Volpato et al., 2021) (Figures 2D,E, respectively), taking inspiration from previous reports on photoelectrochemical water oxidation (Ronconi et al., 2015; Kirner and Finke, 2017; Bonchio et al., 2019; Volpato et al., 2020). In both cases, the systems were proposed for the oxidation of benzyl alcohol in the presence of a redox mediator and a base in the acetonitrile solution, reaching J of 18 and 90 μA/cm^2^, respectively. In the case of the Ku-quinone dye, it is worth mentioning the high oxidative power of the excited state (>2 V *vs*. NHE) that drives the direct oxidation of the species in the solution followed by electron injection into the semiconductor from its radical anion, that was characterized by transient spectroscopy (Volpato et al., 2020; Volpato et al., 2021). In this case, the ability of the dye to exchange protons in concomitance with electrons could open a new perspective by offering the possibility of performing the photoelectrochemical oxidation of alcohols in the absence of a catalyst, thus enlarging the arsenal of organic dyes usable in the design of photoanodes for selective oxidation.

## Conclusion and Future Perspectives

The systems presented in this mini-review demonstrate the great potential of hybrid molecular-based photoanodes when applied to photoredox reactions to replace water oxidation in the production of solar fuels. While the research in this field was pioneered with Ru-based photosensitizers, recent contributions have demonstrated the great potential of noble metal–free and organic photosensitizers. Because of milder operative conditions required with respect to photoelectrochemical water oxidation, it is expected that the pool of organic photosensitizers for such applications will enlarge soon. Considering the scarcity and cost of ruthenium, this observation is particularly encouraging, although definitive conclusions cannot be drawn because of the very limited number of systems reported so far. Almost all discussed systems require an externally applied bias, which has to be provided from external energy sources. Thus, the development of more active and stable sensitizers and unbiased systems (e.g., via tandem approaches) will probably constitute the development of this field in the near future. The biggest challenge, however, is probably the application of these systems to more attractive reactions, such as the selective oxidative activation of C–H bonds. So far, these processes have been pioneered with semiconductor-based photoanodes such as BiVO_4_ (Li et al., 2017) and WO_3_ (Tateno et al., 2018), and for the oxidation of benzylic C–H in tetralin and of aliphatic C–H bonds in cyclohexane. In this regard, molecular components offer the possibility to tune the system according to the mechanistic requirement of the target transformation. Molecular photocatalysts have been already proposed for the homogenous hydroxylation of aromatic C–H, proceeding *via* one-electron oxidation of the aromatic ring followed by water insertion (Fukuzumi et al., 2019). However, for inactivated aliphatic C–H bonds, the one-electron oxidation usually requires high potentials (>2.5 V *vs*. SCE) (Baran 2017). Therefore, hydroxylation of such C–H bonds should consider more favorable pathways through a formal hydrogen atom abstraction as the first step, where the bond dissociation free energy (BDFE) should be considered the key descriptor of reactivity (BDFE of C–H bonds stands in the range 70–110 kcal/mol, (Agarwal et al., 2022)). Within this scenario, the use of transition metal catalysts enabling the formation of high-valent metal-oxo species could be envisaged.

## References

[B1] Agarwal R. G. Coste S. C. Groff B. D. Heuer A. M. Noh H. Parada G. A. (2022). Free Energies of Proton-Coupled Electron Transfer Reagents and Their Applications. Chem. Rev. 122 (1), 1–49. 10.1021/acs.chemrev.1c00521 34928136 PMC9175307

[B2] Agosti A. Natali M. Amirav L. Bergamini G. (2020). Towards Solar Factories: Prospects of Solar‐to‐Chemical Energy Conversion Using Colloidal Semiconductor Photosynthetic Systems. ChemSusChem 13 (18), 4894–4899. 10.1002/cssc.202001274 32809266

[B3] Antón-García D. Edwardes Moore E. Bajada M. A. Eisenschmidt A. Oliveira A. R. Pereira I. A. C. (2022). Photoelectrochemical Hybrid Cell for Unbiased CO2 Reduction Coupled to Alcohol Oxidation. Nat. Synth. 1 (1), 77–86. 10.1038/s44160-021-00003-2

[B4] Badgurjar D. Shan B. Nayak A. Wu L. Chitta R. Meyer T. J. (2020). Electron-Withdrawing Boron Dipyrromethene Dyes as Visible Light Absorber/Sensitizers on Semiconductor Oxide Surfaces. ACS Appl. Mat. Interfaces 12 (6), 7768–7776. 10.1021/acsami.9b20167 31961645

[B5] Balzani V. Credi A. Venturi M. (2008). Photochemical Conversion of Solar Energy. ChemSusChem 1 (1-2), 26–58. 10.1002/cssc.200700087 18605661

[B6] Berardi S. Drouet S. Francàs L. Gimbert-Suriñach C. Guttentag M. Richmond C. (2014). Molecular Artificial Photosynthesis. Chem. Soc. Rev. 43 (22), 7501–7519. 10.1039/C3CS60405E 24473472

[B7] Bonchio M. Syrgiannis Z. Burian M. Marino N. Pizzolato E. Dirian K. (2019). Hierarchical Organization of Perylene Bisimides and Polyoxometalates for Photo-Assisted Water Oxidation. Nat. Chem. 11 (2), 146–153. 10.1038/s41557-018-0172-y 30510216

[B8] Bruggeman D. F. Bakker T. M. A. Mathew S. Reek J. N. H. (2021a). Redox‐Mediated Alcohol Oxidation Coupled to Hydrogen Gas Formation in a Dye‐Sensitized Photosynthesis Cell. Chem. Eur. J. 27 (1), 218–221. 10.1002/chem.202003306 32902899 PMC7839774

[B9] Bruggeman D. F. Mathew S. Detz R. J. Reek J. N. H. (2021b). Comparison of Homogeneous and Heterogeneous Catalysts in Dye-Sensitised Photoelectrochemical Cells for Alcohol Oxidation Coupled to Dihydrogen Formation. Sustain. Energy Fuels 5 (22), 5707–5716. 10.1039/D1SE01275D 34912969 PMC8577521

[B10] Butburee T. Chakthranont P. Phawa C. Faungnawakij K. (2020). Beyond Artificial Photosynthesis: Prospects on Photobiorefinery. ChemCatChem 12 (7), 1873–1890. 10.1002/cctc.201901856

[B11] Casadevall C. (2022). Heterogenization of Molecular Water Oxidation Catalysts in Electrodes for (Photo)Electrochemical Water Oxidation. Water 14 (3), 371. 10.3390/w14030371

[B12] Concepcion J. J. House R. L. Papanikolas J. M. Meyer T. J. (2012). Chemical Approaches to Artificial Photosynthesis. Proc. Natl. Acad. Sci. U.S.A. 109 (39), 15560–15564. 10.1073/pnas.1212254109 23019352 PMC3465377

[B13] Cui Y. Goes S. L. Stahl S. S. (2021). “Sequential Oxidation-Depolymerization Strategies for Lignin Conversion to Low Molecular Weight Aromatic Chemicals,” in Advances in Inorganic Chemistry. Editors Ford P. C. van Eldik R. (Academic Press), 99–136. 10.1016/bs.adioch.2021.02.003

[B14] Eftekhari A. Babu V. J. Ramakrishna S. (2017). Photoelectrode Nanomaterials for Photoelectrochemical Water Splitting. Int. J. Hydrogen Energy 42 (16), 11078–11109. 10.1016/j.ijhydene.2017.03.029

[B15] Field C. B. Behrenfeld M. J. Randerson J. T. Falkowski P. (1998). Primary Production of the Biosphere: Integrating Terrestrial and Oceanic Components. Science 281 (5374), 237–240. 10.1126/science.281.5374.237 9657713

[B16] Fukuzumi S. Lee Y. M. Nam W. (2019). Photocatalytic Oxygenation Reactions Using Water and Dioxygen. ChemSusChem 12 (17), 3931–3940. 10.1002/cssc.201901276 31250964

[B17] Hennessey S. Farràs P. (2018). Production of Solar Chemicals: Gaining Selectivity with Hybrid Molecule/semiconductor Assemblies. Chem. Commun. 54 (50), 6662–6680. 10.1039/C8CC02487A 29808196

[B18] Hong D. Mandal S. Yamada Y. Lee Y.-M. Nam W. Llobet A. (2013). Water Oxidation Catalysis with Nonheme Iron Complexes under Acidic and Basic Conditions: Homogeneous or Heterogeneous? Inorg. Chem. 52 (16), 9522–9531. 10.1021/ic401180r 23895380

[B19] Inoue H. Shimada T. Kou Y. Nabetani Y. Masui D. Takagi S. (2011). The Water Oxidation Bottleneck in Artificial Photosynthesis: How Can We Get through it? an Alternative Route Involving a Two-Electron Process. ChemSusChem 4 (2), a–n. 10.1002/cssc.201000385 21271684

[B20] Jiang J. Sherman B. D. Zhao Y. He R. Ghiviriga I. Alibabaei L. (2017). Polymer Chromophore-Catalyst Assembly for Solar Fuel Generation. ACS Appl. Mat. Interfaces 9 (23), 19529–19534. 10.1021/acsami.7b05173 28545297

[B21] Kampouri S. Stylianou K. C. (2019). Dual-Functional Photocatalysis for Simultaneous Hydrogen Production and Oxidation of Organic Substances. ACS Catal. 9 (5), 4247–4270. 10.1021/acscatal.9b00332

[B55] Kawamata Y. Yan M. Liu Z. Bao D.-H. Chen J. Starr J. T. (2017). Scalable, Electrochemical Oxidation of Unactivated C‐H Bonds. J. Am. Chem. Soc. 139 (22), 7448–7451. 10.1021/jacs.7b03539 28510449 PMC5465511

[B22] Kim S. Kim K. H. Oh C. Zhang K. Park J. H. (2022). Artificial Photosynthesis for High‐value‐added Chemicals: Old Material, New Opportunity. Carbon Energy 4 (1), 21–44. 10.1002/cey2.159

[B23] Kirner J. T. Finke R. G. (2017). Sensitization of Nanocrystalline Metal Oxides with a Phosphonate-Functionalized Perylene Diimide for Photoelectrochemical Water Oxidation with a CoOx Catalyst. ACS Appl. Mat. Interfaces 9 (33), 27625–27637. 10.1021/acsami.7b05874 28727440

[B24] Lewis N. S. (2016). Research Opportunities to Advance Solar Energy Utilization. Science 351 (6271), aad1920. 10.1126/science.aad1920 26798020

[B25] Lhermitte C. R. Sivula K. (2019). Alternative Oxidation Reactions for Solar-Driven Fuel Production. ACS Catal. 9 (3), 2007–2017. 10.1021/acscatal.8b04565

[B26] Li S. Li Z.-J. Yu H. Sytu M. R. Wang Y. Beeri D. (2020). Solar-Driven Lignin Oxidation via Hydrogen Atom Transfer with a Dye-Sensitized TiO2 Photoanode. ACS Energy Lett. 5 (3), 777–784. 10.1021/acsenergylett.9b02391

[B27] Li T. Kasahara T. He J. Dettelbach K. E. Sammis G. M. Berlinguette C. P. (2017). Photoelectrochemical Oxidation of Organic Substrates in Organic Media. Nat. Commun. 8 (1), 390. 10.1038/s41467-017-00420-y 28855502 PMC5577226

[B28] Luo H. Weeda E. P. Alherech M. Anson C. W. Karlen S. D. Cui Y. (2021). Oxidative Catalytic Fractionation of Lignocellulosic Biomass under Non-alkaline Conditions. J. Am. Chem. Soc. 143 (37), 15462–15470. 10.1021/jacs.1c08635 34498845 PMC8487257

[B29] Macchioni A. (2019). The Middle‐Earth between Homogeneous and Heterogeneous Catalysis in Water Oxidation with Iridium. Eur. J. Inorg. Chem. 2019 (1), 7–17. 10.1002/ejic.201800798

[B30] Mazzeo A. Santalla S. Gaviglio C. Doctorovich F. Pellegrino J. (2021). Recent Progress in Homogeneous Light-Driven Hydrogen Evolution Using First-Row Transition Metal Catalysts. Inorganica Chim. Acta 517, 119950. 10.1016/j.ica.2020.119950

[B31] Moore G. F. Blakemore J. D. Milot R. L. Hull J. F. Song H.-e. Cai L. (2011). A Visible Light Water-Splitting Cell with a Photoanode Formed by Codeposition of a High-Potential Porphyrin and an Iridium Water-Oxidation Catalyst. Energy Environ. Sci. 4 (7), 2389–2392. 10.1039/C1EE01037A

[B32] Nikoloudakis E. Pati P. B. Charalambidis G. Budkina D. S. Diring S. Planchat A. (2021). Dye-Sensitized Photoelectrosynthesis Cells for Benzyl Alcohol Oxidation Using a Zinc Porphyrin Sensitizer and TEMPO Catalyst. ACS Catal. 11 (19), 12075–12086. 10.1021/acscatal.1c02609

[B33] Nwosu U. Wang A. Palma B. Zhao H. Khan M. A. Kibria M. (2021). Selective Biomass Photoreforming for Valuable Chemicals and Fuels: A Critical Review. Renew. Sustain. Energy Rev. 148, 111266. 10.1016/j.rser.2021.111266

[B34] Orbelli Biroli A. Tessore F. Di Carlo G. Pizzotti M. Benazzi E. Gentile F. (2019). Fluorinated ZnII Porphyrins for Dye-Sensitized Aqueous Photoelectrosynthetic Cells. ACS Appl. Mat. Interfaces 11 (36), 32895–32908. 10.1021/acsami.9b08042 31429275

[B35] Pho T. V. Sheridan M. V. Morseth Z. A. Sherman B. D. Meyer T. J. Papanikolas J. M. (2016). Efficient Light-Driven Oxidation of Alcohols Using an Organic Chromophore-Catalyst Assembly Anchored to TiO2. ACS Appl. Mat. Interfaces 8 (14), 9125–9133. 10.1021/acsami.6b00932 27032068

[B36] Poddutoori P. K. Thomsen J. M. Milot R. L. Sheehan S. W. Negre C. F. A. Garapati V. K. R. (2015). Interfacial Electron Transfer in Photoanodes Based on Phosphorus(v) Porphyrin Sensitizers Co-deposited on SnO2 with the Ir(III)Cp* Water Oxidation Precatalyst. J. Mat. Chem. A 3 (7), 3868–3879. 10.1039/C4TA07018F

[B37] Qi M.-Y. Conte M. Anpo M. Tang Z.-R. Xu Y.-J. (2021). Cooperative Coupling of Oxidative Organic Synthesis and Hydrogen Production over Semiconductor-Based Photocatalysts. Chem. Rev. 121 (21), 13051–13085. 10.1021/acs.chemrev.1c00197 34378934

[B38] Reisner E. (2019). When Does Organic Photoredox Catalysis Meet Artificial Photosynthesis? Angew. Chem. Int. Ed. 58 (12), 3656–3657. 10.1002/anie.201814692 30701635

[B39] Ronconi F. Syrgiannis Z. Bonasera A. Prato M. Argazzi R. Caramori S. (2015). Modification of Nanocrystalline WO3 with a Dicationic Perylene Bisimide: Applications to Molecular Level Solar Water Splitting. J. Am. Chem. Soc. 137 (14), 4630–4633. 10.1021/jacs.5b01519 25837588

[B40] Schultz D. M. Yoon T. P. (2014). Solar Synthesis: Prospects in Visible Light Photocatalysis. Science 343 (6174), 1239176. 10.1126/science.1239176 24578578 PMC4547527

[B41] Song W. Vannucci A. K. Farnum B. H. Lapides A. M. Brennaman M. K. Kalanyan B. (2014). Visible Light Driven Benzyl Alcohol Dehydrogenation in a Dye-Sensitized Photoelectrosynthesis Cell. J. Am. Chem. Soc. 136 (27), 9773–9779. 10.1021/ja505022f 24933178

[B42] Tang J.-H. Sun Y. (2020). Visible-light-driven Organic Transformations Integrated with H2 Production on Semiconductors. Mat. Adv. 1 (7), 2155–2162. 10.1039/D0MA00327A

[B43] Tateno H. Iguchi S. Miseki Y. Sayama K. (2018). Photoelectrochemical C−H Bond Activation of Cyclohexane Using a WO3 Photoanode and Visible Light. Angew. Chem. Int. Ed. 57 (35), 11238–11241. 10.1002/anie.201805079 30059182

[B44] Toe C. Y. Tsounis C. Zhang J. Masood H. Gunawan D. Scott J. (2021). Advancing Photoreforming of Organics: Highlights on Photocatalyst and System Designs for Selective Oxidation Reactions. Energy Environ. Sci. 14 (3), 1140–1175. 10.1039/D0EE03116J

[B45] Treadway J. A. Moss J. A. Meyer T. J. (1999). Visible Region Photooxidation on TiO2 with a Chromophore−Catalyst Molecular Assembly. Inorg. Chem. 38 (20), 4386–4387. 10.1021/ic990466m 11671146

[B46] Uekert T. Pichler C. M. Schubert T. Reisner E. (2021). Solar-driven Reforming of Solid Waste for a Sustainable Future. Nat. Sustain. 4 (5), 383–391. 10.1038/s41893-020-00650-x

[B47] Volpato G. A. Colusso E. Paoloni L. Forchetta M. Sgarbossa F. Cristino V. (2021). Artificial Photosynthesis: Photoanodes Based on Polyquinoid Dyes onto Mesoporous Tin Oxide Surface. Photochem. Photobiol. Sci. 20 (10), 1243–1255. 10.1007/s43630-021-00097-9 34570354

[B48] Volpato G. A. Marasi M. Gobbato T. Valentini F. Sabuzi F. Gagliardi V. (2020). Photoanodes for Water Oxidation with Visible Light Based on a Pentacyclic Quinoid Organic Dye Enabling Proton-Coupled Electron Transfer. Chem. Commun. 56 (15), 2248–2251. 10.1039/C9CC09805D 31993616

[B49] Wu H.-L. Li X.-B. Tung C.-H. Wu L.-Z. (2018). Recent Advances in Sensitized Photocathodes: From Molecular Dyes to Semiconducting Quantum Dots. Adv. Sci. 5 (4), 1700684. 10.1002/advs.201700684 PMC5908380 29721417

[B50] Yamamoto M. Nishizawa Y. Chábera P. Li F. Pascher T. Sundström V. (2016). Visible Light-Driven Water Oxidation with a Subporphyrin Sensitizer and a Water Oxidation Catalyst. Chem. Commun. 52 (94), 13702–13705. 10.1039/C6CC07877J 27819083

[B51] Yuan Y. Jin N. Saghy P. Dube L. Zhu H. Chen O. (2021). Quantum Dot Photocatalysts for Organic Transformations. J. Phys. Chem. Lett. 12 (30), 7180–7193. 10.1021/acs.jpclett.1c01717 34309389

[B52] Zhang B. Sun L. (2019). Artificial Photosynthesis: Opportunities and Challenges of Molecular Catalysts. Chem. Soc. Rev. 48 (7), 2216–2264. 10.1039/C8CS00897C 30895997

[B53] Zhou X. Li F. Li X. Li H. Wang Y. Sun L. (2015). Photocatalytic Oxidation of Organic Compounds in a Hybrid System Composed of a Molecular Catalyst and Visible Light-Absorbing Semiconductor. Dalton Trans. 44 (2), 475–479. 10.1039/C4DT02945C 25407102

[B54] Zhuang J.-L. Shen Y.-M. Xue Y. Yan M. Cheng H. Chen Z. (2020). Electrochemical Deposition of Perylene-Based Thin Films from Aqueous Solution and Studies of Visible-Light-Driven Oxidation of Alcohols. ACS Appl. Energy Mat. 3 (9), 9098–9106. 10.1021/acsaem.0c01475

